# Anemia in patients ≥ 75 years with metastatic clear cell renal cell carcinoma: an important poor prognostic factor in the international metastatic renal cell carcinoma database consortium model

**DOI:** 10.1186/s12894-024-01403-0

**Published:** 2024-01-11

**Authors:** Ryuichi Mizuno, Yota Yasumizu, Nobuyuki Tanaka, Toshikazu Takeda, Shinya Morita, Kazuhiro Matsumoto, Takeo Kosaka, Hiroshi Asanuma, Mototsugu Oya

**Affiliations:** Department of Urology, Keio University School of Medicine, Tokyo, 1608582 Japan

**Keywords:** Kidney cancer, Prognosis, Elderly

## Abstract

**Background:**

Due to an increase in life expectancy, the incidence of metastatic renal cell carcinoma (mRCC) in patients aged ≥75 years has been increasing. In this study we investigated the characteristics before treatment and the outcomes of systemic therapies for patients aged ≥75 years with mRCC and compared the results with those for patients aged < 75 years in order to determine whether differences in age influenced survival.

**Methods:**

A total of 206 consecutive Japanese patients with mRCC, including 47 patients aged ≥75 years, who received systemic therapy were included. Clinical data from medical records were retrieved and analyzed retrospectively. Survival analyses were determined using a Kaplan–Meier method, and analyzed with a log-rank test.

**Results:**

Elderly patients categorized as favorable risk group based on the International Metastatic RCC Database Consortium (IMDC) stratification system were significantly lower. Among IMDC risk factors, the rate of anemia was significantly higher in elderly patients. No statistically significant benefit in progression free survival for first and second line treatment was observed, whereas improvements in overall survival as well as cancer specific survival were seen in patients aged < 75 years.

**Conclusions:**

For mRCC patients aged ≥75 years, a higher proportion of base line anemia, which resulted in higher rates of IMDC intermediate/poor risk, would be responsible for shorter OS/CSS. Furthermore, mRCC patients aged ≥75 years tend to receive BSC instead of second line active treatment. Overcoming under-treatment in elderly patients might help to prolong survival in mRCC.

**Supplementary Information:**

The online version contains supplementary material available at 10.1186/s12894-024-01403-0.

## Introduction

Renal cell carcinoma (RCC) is the most common malignant tumor of the kidneys, accounting for up to 90% of all renal cancers. In the past decade, the incidence of RCC, including the number of patients ≥75 years, has been increasing annually. Clear cell RCC (ccRCC) is very prevalent and mutations in the *von Hippel-Lindau* (VHL) gene are detected in up to 80% of cases, which results in the constitutive activation of the downstream angiogenesis pathway [1]. The standard curative treatment for localized RCC is surgical excision, and there are a number of patients with de novo or recurrent metastatic RCC (mRCC). With the demonstrated efficacy and safety of targeted inhibitors of vascular endothelial growth factor (VEGF) receptor and mammalian target of rapamycin (mTOR), a larger percentage of patients with mRCC now receive systemic therapy. Furthermore, the introduction of immune checkpoint inhibitors (ICIs) has revolutionized first-line and subsequent treatments for mRCC. As a result, median overall survival (OS) has increased to more than 4 years in recent trials [2–5]. However, optimal management has not yet been established for elderly mRCC patients.

The elderly are considered to be ≥65 years; however, there is no clear medical or biological evidence to support this definition. Based on analyses of various data on the physical and psychological health of elderly individuals, the most recent classification for elderly is ≥75 years [6]. Due to an increase in life expectancy, more patients ≥75 years are being diagnosed with mRCC and considered for active treatment. The incidence of chronic health conditions significantly increases with aging. Systemic therapy for elderly patients may be more challenging and complex because of the elevated risk of serious adverse drug reactions. Therefore, the anticipated benefits of systemic therapy as well as possible adverse events in the elderly need to be evaluated during decision-making for a treatment plan.

In the present study, we investigated the characteristics of mRCC patients ≥75 years before treatment and the outcomes of systemic therapy. We then compared the results obtained with those in patients < 75 years to clarify whether age differences affect survival.

## Patients and methods

### Patient selection and data collection

In this retrospective study, we collected clinical information on 206 consecutive Japanese patients with mRCC, including 47 patients ≥75 years, who received systemic therapy at Keio University Hospital between 2008 and 2022. All patients were pathologically diagnosed with ccRCC. Ethical approval was obtained from the local Ethics Committee of Keio University in view of the retrospective nature of the study (Approval No-20130425). All procedures were performed in accordance with the 1964 Helsinki Declaration and its later amendments or comparable ethical standards. Given the retrospective nature of this cohort study, informed consent was acquired via an opt-out mechanism on the Keio University website (http://www.keio-urology.jp/).

All patients were classified into three different categories (favorable risk group (Fav), intermediate risk group (Int), and poor risk group (Por)) based on the International Metastatic RCC Database Consortium (IMDC) risk model by utilizing baseline clinical parameters [7]. All patients underwent a medical examination and blood test at baseline and then every 2–4 weeks during systemic therapy. Computed tomography (CT) or magnetic resonance imaging (MRI) was performed for a radiographic evaluation every 3–6 months. Head CT/MRI and bone scintigraphy were conducted when brain or bone metastasis was clinically suspected. Radiologic images were reviewed and the sum of the longest diameter of each target lesion was measured and assessed according to the response evaluation criteria in solid tumors (RECIST) version 1.1 for each CT and MRI image [8]. Clinical data from medical records, including age, sex, the locations of metastatic sites, the types of first- and second-line systemic therapies, IMDC risk factors, and baseline C reactive protein (CRP) levels, were retrieved and retrospectively analyzed.

### Statistical analysis

Differences in several factors between the two groups were analyzed by the chi-squared and Wilcoxon tests. The effects of baseline hemoglobin on the estimated glomerular filtration rate (eGFR) and age were evaluated using a linear regression model and Spearman’s rank correlation. Progression-free survival (PFS) was defined as the length of time between the initiation of systemic therapy and the progression of mRCC. OS and cancer-specific survival (CSS) were defined as the time from the initiation of first-line systemic therapy to death from any cause and mRCC, respectively. PFS, OS, and CSS were examined using the Kaplan–Meier method and Log-rank test. Statistical analyses was performed using the SPSS version 26 statistical software package (IBM-SPSS Inc., Tokyo, Japan) and *p* values < 0.05 were considered to be significant. Propensity score matching was used to adjust a treatment effect for a baseline covariate. In the present study, we matched and balanced patients ≥75 years and those < 75 years in pairs for the IMDC risk group (fav/int vs poor) and anemia. The matching and balancing of empirical distributions were performed by logistic regression analyses [9]. Propensity score matching made the ≥75 years and < 75 years groups homogenous in terms of the IMDC risk group distribution (fav vs int/poor) or anemia. Therefore, the results obtained provided less bias in the evaluation of the effects of aging on OS/CSS.

## Results

### Patient characteristics

Among the 206 patients examined, 47 (22.8%) were ≥ 75 years and 159 (77.2%) were < 75 years. Table 1 shows clinical parameters and treatment exposure profiles according to age. Nephrectomy was performed in 136/159 cases for those < 75 years, compared to 37/47 cases for those ≥75 years. A linear regression model revealed a significant correlation between baseline hemoglobin and eGFR in patients ≥75 years (*p* = 0.0059), as well as in the entire cohort (*p* = 0.0111, Supplementary Fig. 1A). Spearman’s rank correlation indicated that hemoglobin was associated with eGFR (r = 0.2405, *p* = 0.0005). Additionally, the model indicated a correlation between baseline hemoglobin and age in the entire cohort (*p* = 0.0004, Supplementary Fig. 1B); however, no correlation was observed in patients ≥75 years.

**Table 1:** Patient characteristics

Parameters	≥75 years	< 75 years	*p* value
Gender (%)			N.S
-Male/Female	66/34	82.4/17.6	
IMDC classification (%)			0.0241
-Fav	14.9	30.8	
-Int/Poor	85.1	69.2	
IMDC factors (%)			
-Anemia	61.7	39	0.006
-KPS < 80	23.4	11.9	N.S
-Corrected calcium	6.4	5	N.S
-Within 1 year	40	44	N.S
-Platelet count	8.5	11.9	N.S
-Neutrophil count	42.6	30.2	N.S
CRP (mg/dl)	1.1	1.4	N.S
eGFR(mL/min/1.73m^2^)	38.9	52.6	<0.0001

*IMDC* International Metastatic RCC Database Consortium, *KPS* KarnofskyPerformance Status *CRP* C-reactive protein, *eGFR* estimated glomerular filtration rate, *N.S* not significant

### Distribution of IMDC risk factors in patients ≥75 years and in those < 75 years

The percentage of patients categorized into the favorable risk group based on the IMDC stratification system was significantly lower among patients ≥75 years (14.9%) than among those < 75 years (30.8%, *p* = 0.0241, Table 1). We examined each risk factor for IMDC and found that the rate of anemia was significantly higher in patients ≥75 years (61.7%) than in those < 75 years (39.0%, *p* = 0.006), whereas no significant differences were observed in the remaining five factors (Table 1).

### Systemic therapies and oncological outcomes in the overall population

In total, 18 (8.7%), 147 (71.4%), 31 (15.0%), and 10 (4.9%) patients received cytokine, VEGF-targeted, ICI-based combination, and mTOR- targeted therapies as first-line treatment, respectively. Nine patients (4.4%) achieved a complete response (CR), 44 (21.4%) a partial response (PR), 118 (57.2%) stable disease (SD), and 27 (13.1%) progressive disease (PD), whereas 8 (3.9%) were not evaluable. At the time of the analysis, 185 patients (89.8%) had discontinued first-line therapies, 124 had moved to second-line therapies (VEGF-targeted 76, mTOR- targeted 27, and ICI-based monotherapy 21), and 49 had moved to best supportive care (BSC). After a median follow-up of 39.3 months after treatment initiation, median PFS for first-line systemic therapy was 19.9 months (95% confidence interval (CI): 14.1–25.4) and that for second line systemic therapy was 11.0 months (95% CI: 8.9–20.8). Median OS and CSS were 54.3 months (95% CI: 45.4–83.4) and 56.8 months (95% CI: 45.6–84.5), respectively.

### Systemic therapies and oncological outcomes in patients ≥75 years and in those < 75 years

**Fig. 1:**
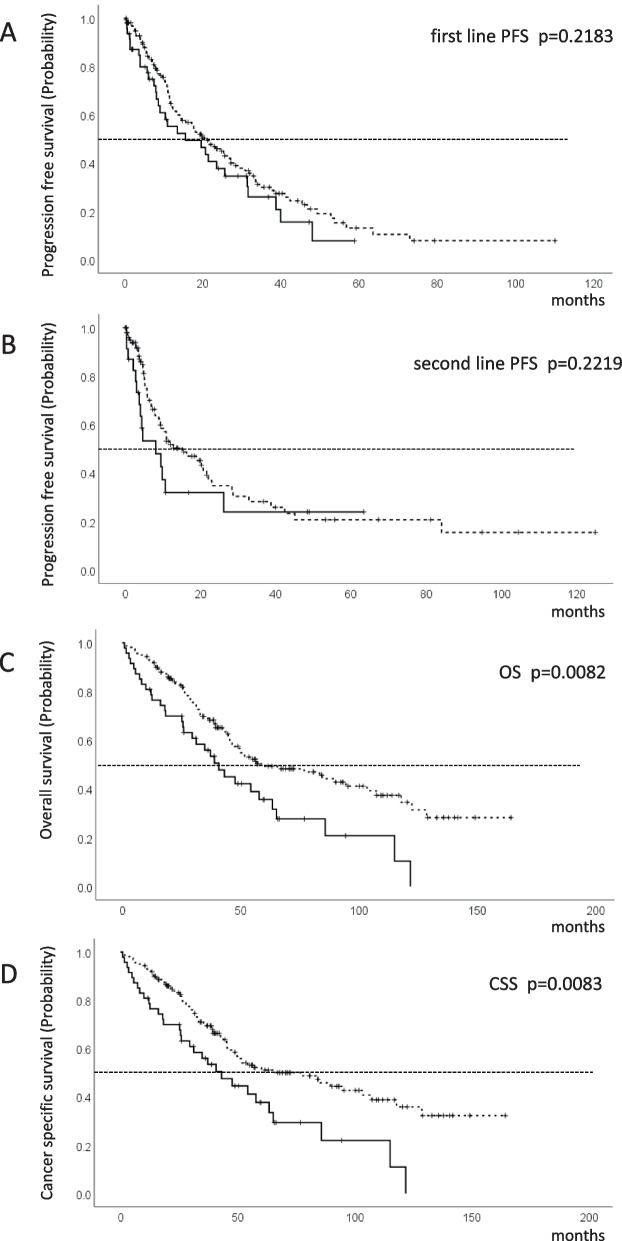
Kaplan-Meier curves showing PFS, OS, and CSS. Kaplan-Meier curves for (**A**) PFS for first-line systemic therapy, (**B**) PFS for second-line systemic therapy, (**C**) OS from the initiation of first-line systemic therapy, and (**D**) CSS from the initiation of first-line systemic therapy in mRCC patients ≥75 years and those < 75 years. + censored case

In patients ≥75 years and those < 75 years, 4/14 (8.5/8.8%), 34/113 (72.3/71.2%), 4/27 (8.6/16.9%), and 5/5 (10.6/3.1%) patients received cytokine, VEGF-targeted, ICI-based combination, and mTOR- targeted therapies, respectively, as first-line treatments. In patients ≥75 years and those < 75 years, 0/9 patients (0.0/5.7%) achieved CR, 6/38 (12.8/23.9%) PR, 30/88 (63.8/55.3%) SD, and 8/19 (17.0/11.9%) PD, respectively. At the time of analysis, progression on first-line treatment occurred in 99/159 cases for patients < 75 years, compared to 30/47 cases for those ≥75 years. At the time of analysis, cancer-specific deaths and deaths from all causes for patients < 75 years were 76/159 cases and 80/159 cases, respectively. In contrast, for those ≥75 years, the corresponding figures were 31/47 cases and 32/47 cases, respectively. Median PFS for first-line therapy was 19.9 (95% CI 14.5–26.8) months in patients < 75 years and 15.6 (95% CI 8.1–25.7) months in those ≥75 years (Log-rank *p* = 0.2183) (Fig. 1a). Median PFS for second-line therapy was 12.8 (95% CI 8.9–22.2) months in patients < 75 years and 8.2 (95% CI 3.0–26.2) months in those ≥75 years (Log-rank *p* = 0.2219) (Fig. 1b). Median OS was 60.2 (95% CI 45.6–103.5) months in patients < 75 years and 40.8 (95% CI 25.7–63.4) months in those ≥75 years (Log-rank *p* = 0.0082) (Fig. 1c). Median CSS was 65.6 (95% CI 46.5–107.3) months in patients < 75 years and 43.2 (95% CI 25.7–63.4) months in those ≥75 years (Log-rank *p* = 0.0083) (Fig. 1d). Therefore, no significant difference were observed for PFS after first- and second-line treatments, whereas OS and CSS were prolonged in patients < 75 years. After propensity score matching based on the IMDC risk group and anemia, 46 and 47 patients remained in each group, respectively. Subsequently, we conducted survival analyses on the propensity score-matched cohort. Using the IMDC risk group as a covariate, we observed no significant differences in OS (log-rank *p* = 0.203) and cancer-CSS (log-rank *p* = 0.172) between patients aged ≥75 years and those < 75 years. Furthermore, when anemia was considered as a covariate, no significant disparities in OS (log-rank *p* = 0.070) and CSS (log-rank *p* = 0.090) were identified between patients aged ≥75 years and those < 75 years.

### Flow of systemic therapy in patients ≥75 years and in those < 75 years

**Fig. 2:**
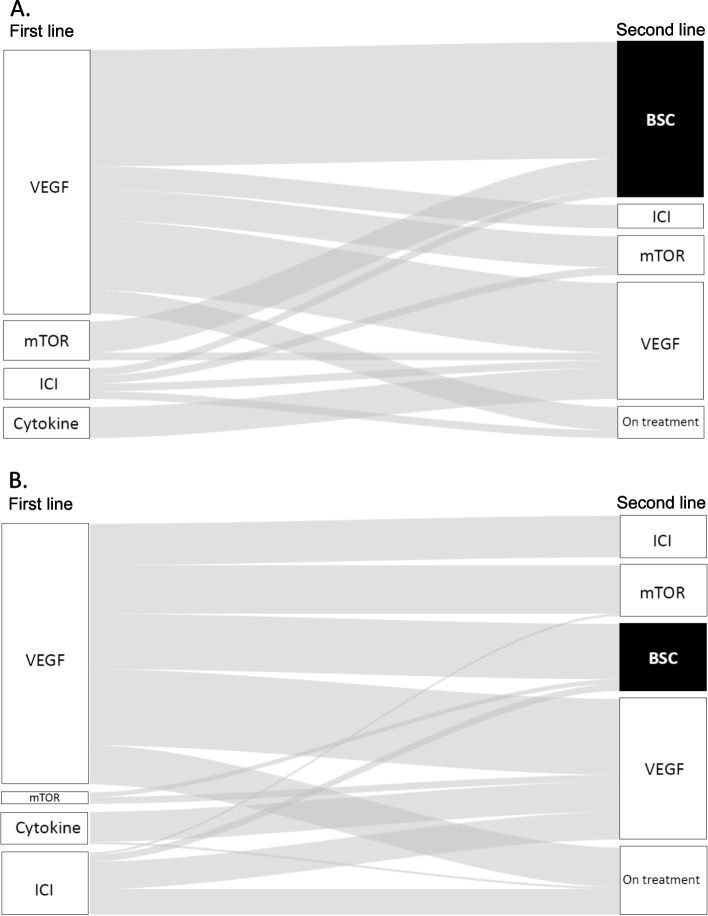
Sankey diagrams showing treatment flow. First- to second-line treatment flow in mRCC patients (**A**) ≥ 75 years and (**B**) < 75 years. Elderly patients were more likely to receive BSC instead of active second-line therapy

Due to the lack of a significant difference in PFS and prolonged OS/CSS in the same cohort, we examined treatment sequences. Figure 2 shows the flow of treatment across the different lines of therapy in a Sankey plot. Among 159 patients < 75 years who received first-line therapies, 143 (89.9%) completed treatment; 102 (71.3%) subsequently moved to second-line therapies, and 41 (28.7%) received BSC. Among the 47 patients ≥75 years who received first-line therapies, 45 (95.7%) completed treatment; 23 (51.2%) then moved to second-line therapies and 22 (48.8%) received BSC.

## Discussion

Previous studies reported that an older age did not affect survival outcomes in mRCC [10–12]. However, this retrospective analysis revealed that OS and CSS were significantly shorter in mRCC patients ≥75 years, despite no significant difference observed in PFS after first- and second-line therapies (Fig. 1a, b). Possible explanations for OS and CSS in patients aged ≥75 years include an unbalanced distribution of patients in the IMDC risk group, a higher prevalence of anemia (Table 1), and a higher percentage of patients receiving BSC instead of second-line systemic therapy (Fig. 2).

We attributed the shorter survival of patients ≥75 years to their higher distribution in the IMDC Int/Por risk group. Among six predefined IMDC parameters, the percentage of patients with anemia was significantly higher among those ≥75 years. In many malignancies, including mRCC, baseline anemia has been shown to have a negative impact on prognosis [7, 13]. Furthermore, several studies have confirmed anemia as a risk factor associated with tumor aggressiveness, resistance to systemic therapy, and declined functional status, leading to poor quality of life [14, 15]. The causes of anemia in cancer patients are diverse and cannot be fully explained by typical triggers such as bone marrow infiltration, blood loss, hemolysis, renal, hepatic, or endocrine disorders, or nutritional deficiencies. The prevalence of anemia, a common hematological condition in cancer patients, increases with age [15]. In this study, anemia emerged as a significant prognostic factor in mRCC patients ≥75 years. In the propensity analysis, which holds higher quality compared to a mere observational study, when focusing solely on anemia as a covariate, there still appears to be a trend indicating worse OS and CSS for those ≥75 years. Among mRCC patients ≥75 years, in addition to anemia from the direct effects of cancers, anemia from non-cancerous disorders may shorten survival. Since anemia is a common complication in chronic kidney disease (CKD), mRCC patients with a previous history of nephrectomy are susceptible to a decline in renal function [16]. As expected, a correlation was observed between anemia and reduced eGFR at baseline in our cohort. Furthermore, baseline eGFR significantly decreased in patients ≥75 years (Table 1). The eGFR starts to decline with normal aging, generally after 30–40 years of age, and the rate of this decline may accelerate after 50–60 years of age [17]. This reduction appears to be a part of the normal physiological process of cellular and organ senescence and is associated with structural changes in the kidneys. However, in mRCC patients with a previous history of nephrectomy and, thus, a decline in renal function, reductions in eGFR may be further accelerated by aging. We speculate that an age-related decline in kidney function contributed to the high prevalence of anemia with CKD in patients ≥75 years and, as such, increased the number of baseline IMDC risk factors.

In several cancers, healthy elderly patients obtain benefits from the same systemic therapy as younger patients; however, under-treatment in elderly patients is a very real issue and is regarded as one of the main reasons for poorer outcomes than in young adults [18]. Possible reasons for the under-treatment of mRCC include patient preferences, treatment costs, and a lack of guidelines for evidence-based decisions for the treatment of elderly patients [19]. With a more detailed understanding of the molecular oncology of this disease, treatment strategies for mRCC have evolved from cytokines to targeted anti-angiogenic agents and ICI in the past few decades. These novel approaches for the treatment of mRCC have resulted in significant advances and longer survival, but have also increased costs for elderly patients and their family. In addition, elderly patients must be prepared to fund transportation to and from appointments and perhaps to a specialist center. After first-line systemic therapy, elderly patients are more likely to hesitate to receive active second-line therapy, which may prolong CSS/OS, because of concerns regarding the loss of physical fitness with disease progression or the development of treatment-related adverse events. Therefore, additional funding is required to support and continue systemic therapy for elderly patients due to their decreasing ability to maintain the activities of daily living.

Several phase 3 studies demonstrated the efficacy of new agents over standard treatment; however, since elderly patients were ineligible to participate in these oncological randomized clinical trials, there is a lack of guidelines for the treatment of these patients. Previous studies reported that elderly patients with mRCC were more prone to toxicity, which resulted in a higher percentage of interrupted treatment. In the present study, no significant differences were observed in the rate of discontinuation of first-line systemic therapies between the two groups. With a lack of evidence, clinicians are uncertain whether all elderly patients may tolerate aggressive systemic therapy or achieve survival benefits. Therefore, standard guidelines for elderly patients with mRCC are warranted.

The limitations of the present study include the sample size examined and its retrospective nature. Further studies are needed to confirm whether active treatment choices are associated with prolonged OS.

## Conclusion

The higher percentage of mRCC patients ≥75 years with baseline anemia, which resulted in a higher rate of IMDC Int/Por, may be responsible for shorter OS/CSS. Furthermore, mRCC patients ≥75 years were more likely to receive BSC instead of second-line active therapies. The prevention of under-treatment in elderly mRCC patients may prolong their survival.

### Supplementary Information

**Additional file 1: Figure S1.** Correlations between age, eGFR, and hemoglobin along with corresponding scatter plots.

## Data Availability

The data that support the findings of this study are not publicly available on ethical ground but are available from the corresponding author with permission from the Keio University School of Medicine Ethics Committee.

## Abbreviations

RCC: Renal cell carcinoma
mRCC: Metastatic renal cell carcinoma
IMDC: International Metastatic RCC Database Consortium
ccRCC: Clear cell renal cell carcinoma
VHL: *von Hippel-Lindau*
VEGF: Vascular endothelial growth factor
mTOR: Mammalian target of rapamycin
ICIs: Immune checkpoint inhibitors
OS: Overall survival
Fav: Favorable risk group
Int: Intermediate risk group
Por: Poor risk group
RECIST: Response evaluation criteria in solid tumors
CRP: C reactive protein
eGFR: Estimated glomerular filtration rate
PFS: Progression-free survival
CSS: Cancer-specific survival
CKD: Chronic kidney disease
