# Supplementary material for: Anemia in patients ≥ 75 years with metastatic clear cell renal cell carcinoma: an important poor prognostic factor in the international metastatic renal cell carcinoma database consortium model
Source: BMC Urol. 2024 Jan 11;24:13. doi: 10.1186/s12894-024-01403-0 (PMC10782570; doi:10.1186/s12894-024-01403-0)

Supplementary Figure 1. Correlations between age, eGFR, and hemoglobin along with corresponding scatter plots.

A linear regression model between baseline hemoglobin and eGFR (A) (p=0.0111), between baseline hemoglobin and age (B) (p=0.0004) in the entire cohort.

A

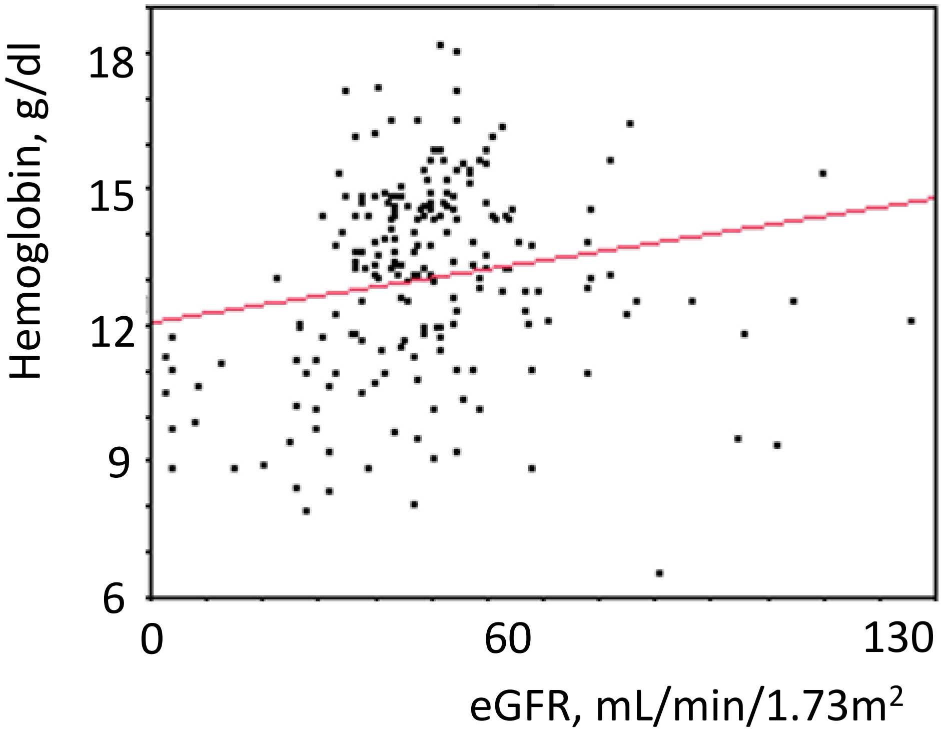

B

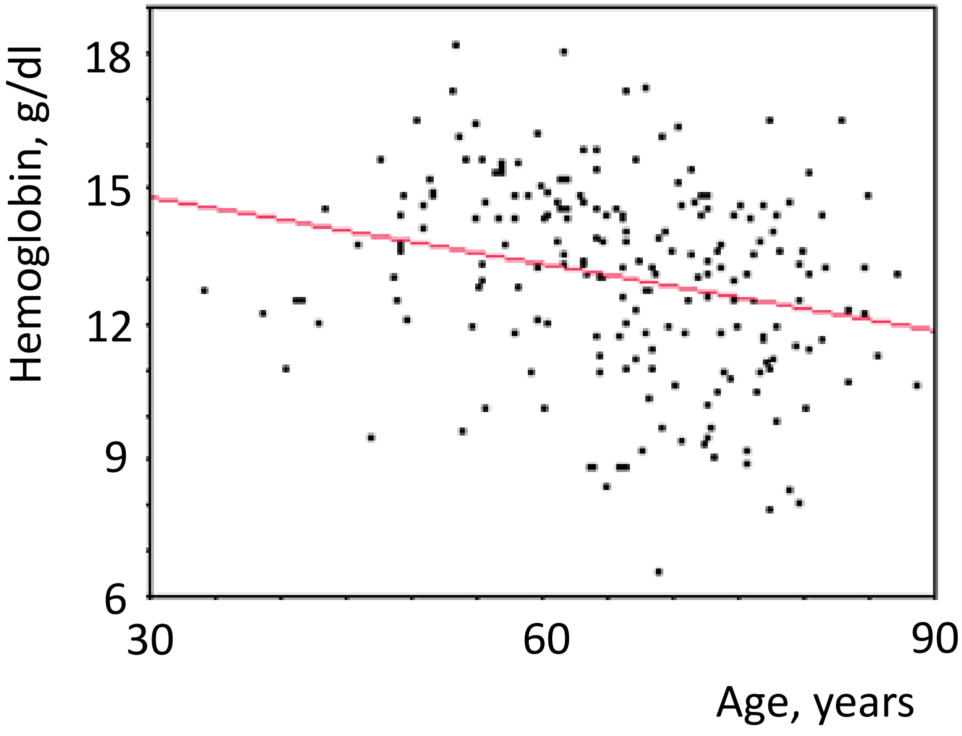

Supplement: Supplementary file 1 — Additional file 1: Figure S1. Correlations between age, eGFR, and hemoglobin along with corresponding scatter plots. [file 12894_2024_1403_MOESM1_ESM.pdf]
